# Comparative Evaluation of Gelatin and HPMC Inhalation Capsule Shells Exposed to Simulated Humidity Conditions

**DOI:** 10.3390/pharmaceutics17070877

**Published:** 2025-07-03

**Authors:** Sabrina Magramane, Nikolett Kállai-Szabó, Dóra Farkas, Károly Süvegh, Romána Zelkó, István Antal

**Affiliations:** 1 Department of Pharmaceutics, Semmelweis University, Hőgyes Str. 7, H-1092 Budapest, Hungary; sabrina.magramane.lfgeb@gmail.com (S.M.); kallai.nikolett@semmelweis.hu (N.K.-S.);; 2 Center for Pharmacology and Drug Research & Development, Semmelweis University, 1085 Budapest, Hungary; 3 Department of Analytical Chemistry, Eötvös Loránd University, Pázmány Péter Sétány 1/a, H-1117 Budapest, Hungary; karoly.suvegh@ttk.elte.hu; 4 University Pharmacy Department of Pharmacy Administration, Semmelweis University, Hőgyes Str. 7-9, H-1092 Budapest, Hungary

**Keywords:** gelatin, HPMC, capsules, dry powder inhalers (DPIs), stability, positron annihilation lifetime spectroscopy (PALS)

## Abstract

**Background/Objectives**: This study investigates the impact of high humidity (25 °C, 75% relative humidity) on gelatin and hydroxypropyl methylcellulose (HPMC) capsules used in dry powder inhalers (DPIs), focusing on moisture dynamics, structural responses, and mechanical performance, with an emphasis on understanding how different capsule types respond to prolonged exposure to humid conditions. **Methods**: Capsules were exposed to controlled humidity conditions, and moisture uptake was measured via thermal analysis. Visual observations of silica bead color changes were performed to assess moisture absorption, while surface wettability was measured using the sessile drop method. Hardness testing, mechanical deformation, and puncture tests were performed to evaluate structural and mechanical changes. Positron annihilation lifetime spectroscopy (PALS) was used to analyze free volume expansion. **Results**: HPMC capsules exhibited rapid moisture uptake, attributed to their lower equilibrium moisture content and ability to rearrange dynamically, preventing brittleness. In contrast, gelatin capsules showed slower moisture absorption but reached higher equilibrium levels, resulting in plasticization and softening. Mechanical testing showed that HPMC capsules retained structural integrity with minimal deformation, while gelatin capsules became softer and exhibited reduced puncture resistance. Structural analysis revealed greater free volume expansion in HPMC capsules, consistent with their amorphous nature, compared with gelatin’s semi-crystalline matrix. **Conclusions**: HPMC capsules demonstrated superior humidity resilience, making them more suitable for protecting moisture-sensitive active pharmaceutical ingredients (APIs) in DPI formulations. These findings underline the importance of appropriate storage conditions, as outlined in the Summary of Product Characteristics, to ensure optimal capsule performance throughout patient use.

## 1. Introduction

Inhalation therapy has a rich historical background, having been used for centuries as a means of delivering medicinal substances to alleviate respiratory ailments. While early methods involved inhaling various medicinal vapors and fumes, significant advancements in inhalation devices have been made over the last few decades [1,2,3]. Among these innovations, dry powder inhalers (DPIs) have emerged as a leading option to manage respiratory diseases such as asthma and chronic obstructive pulmonary disease (COPD) due to their unique advantages and effective drug delivery systems [4,5,6]. Recent advances in microparticle formulations and inhaler technologies further highlight the evolving field of pulmonary drug delivery [7]. DPIs facilitate direct medication delivery to the lungs, enabling rapid absorption and minimizing systemic exposure, which ultimately enhances therapeutic outcomes, patient adherence, and overall quality of life [2,8].

The growing preference for DPIs stems from their propellant-free formulations, which not only address environmental concerns associated with traditional inhalers but also provide greater chemical stability compared with liquid alternatives [5,9,10,11]. Moreover, DPIs are user-friendly, requiring minimal coordination during use, making them suitable for a broad demographic, including children and elderly patients. Their breath-actuated design enhances their usability and effectiveness, as they eliminate the need for additional devices such as spacers [10,12].

Despite these advantages, DPI performance is influenced by various factors, including the physicochemical characteristics of the drug formulations (such as moisture sensitivity), the inhaler design, and the patient’s inhalation technique [12,13]. Furthermore, patient behavior can significantly impact DPI effectiveness. For instance, a study of 738 patients conducted in 2016 found that two-thirds rarely checked inhaler expiry dates, often used them after expiry, and had not received proper storage guidance (from doctors, nurses, or pharmacists) [14]. In particular, improper storage (such as removing capsules from their original packaging) can compromise their integrity and reduce therapeutic efficacy. Exposure of capsules to ambient air outside their blister pack (e.g., in pill boxes) has been shown to reduce the fine particle dose (FPD) by approximately 18% within 24 h [15]. Moreover, storing capsules under accelerated humid conditions (40 °C, 75% RH) resulted in a ~50% reduction in FPD for certain DPIs [16,17]. These findings highlight the critical role of capsule packaging and proper patient handling in maintaining DPI performance, particularly in humid environments. Consistent drug delivery remains a challenge, prompting innovations in device and formulation design.

The effectiveness and reliability of DPIs are closely tied to the properties of their capsule shells, which protect and deliver the powdered medication [18]. Ding et al. found that the capsule type significantly affects the performance in carrier-free formulations, with minor variability observed in the carrier-based ones [19]. The study also highlighted how capsule hardness influences flap detachment during piercing, impacting aerosol performance. These findings emphasize the need for careful capsule selection to optimize inhaled formulation performance.

Buttini et al. also highlighted the importance of capsules in inhalation therapies, describing key criteria for ideal inhalation capsules [10]. These include easy puncturing without excessive shell shedding, attached and open flaps that facilitate powder discharge, and minimal retention of powders. Capsule performance is influenced by factors such as material, moisture content, and lubrication. While hard gelatin capsules have been used for over 30 years, humidity-related instability is one of the primary challenges associated with them, often compromising shell strength and limiting suitable fill materials. This has led to growing interest in alternative capsule materials [20]. Gelatin capsules, in particular, can become brittle with low moisture, posing inhalation risks. Modified capsules with plasticizers and hydroxypropyl methylcellulose (HPMC) capsules have been introduced to enhance stability and aerosolization. HPMC capsules, with lower moisture content (4.5–6.5%) compared with gelatin (13–16%), do not become brittle and are of plant origin. They show superior stability, perform better in puncturing tests, and are less prone to moisture-related issues. Ultimately, the choice between gelatin and HPMC capsules depends on their interaction with specific formulations.

This study investigates the behavior of these two types of DPI capsules—gelatin and HPMC—under prolonged exposure to elevated humidity and controlled temperature. By examining empty shells without any active ingredients, this research aims to assess the impact of moisture on the physical and chemical characteristics of the capsule materials. The specific condition was selected based on real-life observations as well as regulatory precedents and relevance to DPI performance under moisture stress. According to the International Council for Harmonisation of Technical Requirements for Pharmaceuticals for Human Use (ICH), long-term stability studies are typically conducted at 25 °C ± 2 °C/60% RH ± 5% or 30 °C ± 2 °C/65% RH ± 5% for 12 months, with accelerated conditions set at 40 °C ± 2 °C/75% RH ± 5% for 6 months [21]. However, these protocols are intended for general pharmaceutical products and do not account for short-term, device-specific humidity exposures. The FDA’s draft guidance for MDIs and DPIs recommends “in-use” stability testing of opened inhalers at around 30 °C/65% RH to reflect patient use and storage conditions [22]. Because no universal standard exists for DPI moisture stress testing, we selected a 24 h exposure at 25 °C/75% RH to simulate a realistic yet challenging humidity scenario to assess capsule integrity and aerosol performance.

To assess moisture uptake and its effect, several measurements were conducted. These included mass measurements to track moisture absorption over time and visual tracking using humidity-indicating silica beads. Changes in mechanical strength were evaluated through hardness testing, while moisture content was quantified directly through thermal analysis. Surface wettability was measured using the sessile drop method to detect changes in hydrophilicity. Mechanical performance was further examined through horizontal and vertical deformation tests, along with puncture testing, which is critical for evaluating the capsules’ suitability for DPI applications. Additionally, positron annihilation lifetime spectroscopy (PALS) was used, as it is widely recognized as one of the primary methods for assessing the size and distribution of free volumes. It provided insights into the free volume and microstructural changes within the polymer matrices of the capsules, which are not detectable by conventional structural testing methods [23,24].

## 2. Materials and Methods

### 2.1. Materials and Experimental Setup

Size 0 empty hard gelatin and HPMC capsules were used in this study. The hard gelatin capsules (Capsugel^®^ Coni-Snap^®^) and HPMC capsules (Capsugel^®^ Vcaps^®^ Plus) were both obtained from Lonza (Basel, Switzerland). To evaluate the effects of moisture exposure, the capsules were stored in a climate chamber (Memmert Constant climate chamber HPP110ECO, Büchenbach, Germany) at 25 °C and 75% relative humidity (RH) for predefined durations: 30 min, 1 h, 2 h, 4 h, 8 h, and 24 h. For each capsule type (gelatin and HPMC), dry capsules (0 h) were included in the experiments as a reference for comparison.

### 2.2. Mass Measurements for Moisture Uptake

The mass of individual capsules was measured before and after exposure to controlled humidity conditions to determine the extent of the moisture uptake. Capsules were taken directly from their original packaging and weighed as quickly as possible to minimize environmental influence. Following their exposure to the humidity conditions, they were weighed again immediately upon removal to minimize any potential moisture loss. For each time point, including the dry reference (0 h), 20 capsules were weighed individually. The mass difference before and after exposure was later calculated as a percentage to assess moisture absorption. All the mass measurements were performed using an analytical balance (Kern ABJ-NM/ABS-N, Kern & Sohn GmbH, Balingen, Germany).

### 2.3. Visual Observations of Silica Beads Color Change

To visually assess the moisture uptake of gelatin and HPMC capsules, silica gel beads (Kieselgel brown/blue, Merck KGaA, Darmstadt, Germany) were used as an indicator. These beads have an approximate diameter of 1–4 mm and contain cobalt (II) chloride as a humidity indicator, displaying a distinct color change from blue (dry) to brown (moist). Before use, the beads were dried in an oven (AccuDry, ARTEK Systems Corporation, Bothell, WA, USA) at 120 °C for ~2 h to ensure full dehydration. For the reference conditions, dry beads were prepared by oven drying, while wet beads were obtained by immersing them in water until fully saturated. The capsules, filled with dry silica beads, were stored under the study conditions, after which the capsules were opened, and the silica beads were immediately photographed using a digital microscope (Keyence VHX-970F; Keyence Corp., Osaka, Japan) to document the color changes indicative of moisture absorption.

### 2.4. Hardness Testing Procedure

The hardness measurements were performed using a tablet hardness tester (8M, Dr. Schleuniger^®^ Pharmatron, Solothurn, Switzerland). The capsules were placed perpendicularly to the metal walls of the machine, with their domes aligned along the walls. The machine then applied force to the capsule. The measurements for both gelatin and HPMC capsules were recorded for five capsules at each time point. The hardness was determined as the force required to compress the capsule, with results expressed in Newtons (N).

### 2.5. Moisture Analysis Procedure

The moisture content of the capsules was determined by moisture analysis, performed using a SCALTEC SMO 01 moisture analyzer (Scaltec Instruments GmbH, Göttingen, Germany). This analysis was conducted after the capsules were flattened as flattening ensures uniform heating and moisture evaporation across the entire surface of the capsule, which is essential for accurate moisture content measurements. To achieve flattening, each capsule was placed individually in a tablet hardness tester, parallel to the metal walls of the machine, with its length aligned along the walls. The machine then applied force to the capsule, flattening it into a flat, oval shape. After flattening, five capsules were placed in the moisture analyzer at once for each thermal analysis measurement.

The moisture analyzer was set to a temperature of 105 °C. The moisture measurements were taken after exposure to the humid conditions. The analyzer measured the moisture content by determining the weight loss during the heating process. For each time point, three parallel measurements were performed, with five capsules of either gelatin or HPMC tested at once, and the moisture content percentage was directly recorded.

### 2.6. Surface Wettability Measurements (Sessile Drop Method)

The contact angle of both gelatin and HPMC capsules was measured using the sessile drop method. A 100 µL syringe with a fixed needle (Pressure-Lok^®^ Series C-160, Precision Sampling, Baton Rouge, LA, USA) was used to deposit a single drop of purified water (aqua purificata) onto the surface of each capsule. The contact angle was then measured using a digital microscope tilted at 90°. The measurements were performed on 5 capsules per condition for both dry capsules and capsules were stored for 24 h in a climate chamber at 25 °C and 75% RH.

### 2.7. Mechanical Testing Procedures

The mechanical properties of the gelatin and HPMC capsules were assessed through texture analysis using the TexturePro Texture Analyzer (Brookfield CT3-4500, AMETEK Brookfield, Middleborough, MA, USA), equipped with a 4.5 kg load cell, in order to evaluate their response to external forces after being exposed to specific controlled conditions. The tests aimed to characterize the capsules’ structural integrity, deformation behavior, and resistance to puncture to provide insights into how moisture uptake affects their mechanical performance. Three types of mechanical tests were conducted: horizontal deformation, vertical deformation, and puncture tests. Each test was performed on 5 capsules per time point. Their parameters are summarized in Table 1.

**Figure 1:**
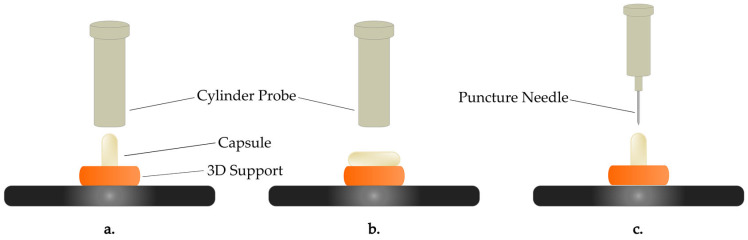
Experimental setup for the mechanical testing of the capsules using texture analysis: (**a**) vertical deformation, (**b**) horizontal deformation, and (**c**) puncture test.

These tests allowed us to investigate key parameters such as the stiffness and the area under the curve (AUC), which reflect changes in the capsules’ mechanical strength and flexibility upon moisture exposure. The capsules were held in position (horizontally or vertically) using a custom 3D-printed support, which was fabricated using an Original Prusa SL1S Speed 3D printer (Prusa Research a.s., Prague, Czech Republic). The support was printed using UV-sensitive Prusament Tough Resin in Prusa Orange. As shown in Figure 1, this support ensured consistent positioning of the capsules during testing in order to minimize variability in the results.

### 2.8. Positron Annihilation Lifetime Spectroscopy (PALS) Analysis

PALS was used to analyze the free volume characteristics of gelatin and HPMC capsules before and after exposure to the controlled humidity conditions. This helped us provide insights on molecular-level structural changes by measuring the lifetime and intensity of ortho-positronium (o-Ps), which correlates with free volume size and distribution in the polymer matrix.

The o-Ps lifetime and intensity were recorded to evaluate moisture-induced modifications in their microstructure. Additionally, the mean positron lifetime was analyzed to assess overall variations in free volume properties.

The positron source used for PALS measurements consisted of carrier-free ^22^NaCl, enclosed between thin Kapton foils (2 mg*cm^−2^). The source had an activity of approximately 5 × 10^5^ Bq. The measurements were carried out using a fast–fast coincidence system, incorporating two BaF_2_ crystal detectors. The system used Philips XP2020Q photomultiplier tubes (Koninklijke Philips N.V., Eindhoven, The Netherlands), and the measuring electronics were based on standard ORTEC units. The system’s time resolution was around 230 ps. For each time point, multiple caspsules were analyzed to ensure reproducibility.

## 3. Results

### 3.1. Mass Changes and Moisture Uptake

The mass of both gelatin and HPMC capsules increased over time due to the moisture uptake in the climate chamber. However, the extent and rate of mass change varied between the two capsule types. Table 2 illustrates that HPMC capsules absorb moisture more rapidly and to a greater extent than gelatin capsules, particularly in the first few hours. Within 30 min, HPMC capsules show a 2.25% mass increase, significantly higher than the 0.78% observed in gelatin capsules. By 1 h, HPMC capsules reach 4.15%, while gelatin capsules increase by 1.12%, showing a lower absorption rate. Over time, HPMC capsules continued to absorb moisture, with a peak of 5.46% at 24 h, though with minor fluctuations between 4 and 8 h. In contrast, gelatin capsules exhibit a steadier moisture uptake, stabilizing at 3.77% after 24 h, following a gradual increase with minimal variation after 8 h.

### 3.2. Visual Observations of Silica Beads Color Change

**Figure 2:**
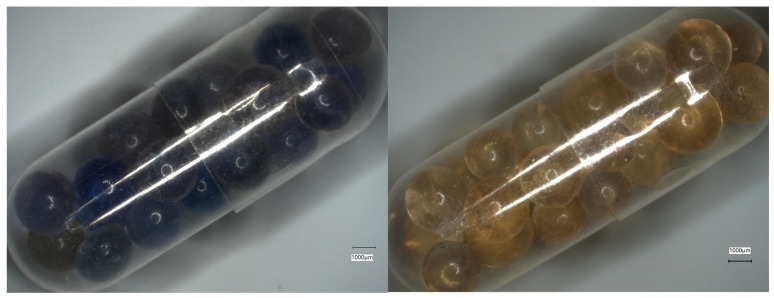
Visual changes in silica beads inside HPMC capsules after 30 min (**left**) and 24 h (**right**) of exposure at 25 °C and 75% relative humidity (RH).

In order to provide a visual representation of the experimental conditions, Figure 2 shows images of HPMC capsules containing the silica beads after 30 min (left) and 24 h (right) of exposure in the climate chamber. These images illustrate how the capsules and beads appeared during storage.

**Figure 3:**
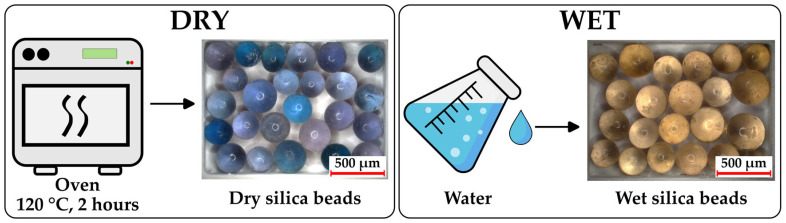
Images of fully dry and fully wet silica beads, used as reference samples to observe the color change from moisture absorption.

**Figure 4:**
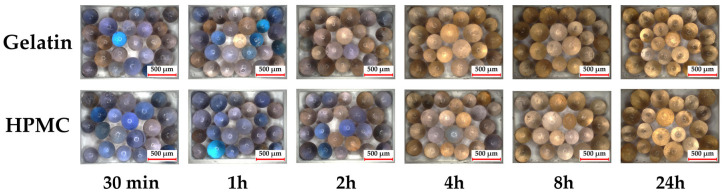
Visual representation of moisture uptake in gelatin and HPMC capsules under 25 °C and 75% RH conditions across the multiple time points.

Figure 3 shows images of the fully dry and fully wet silica beads as a reference, while Figure 4 displays the color changes of the silica beads inside the capsules in the climate chamber over time.

Initially, both gelatin and HPMC capsules contained dried blue silica beads. By 30 min, beads in the gelatin capsule began shifting towards brown, indicating early moisture uptake, while beads in HPMC capsules showed minimal change. After 1 h, gelatin beads exhibited a more pronounced color shift, with some turning brown, while HPMC beads retained mostly blue tones, with only slight brownish hints. At 2 and 4 h, gelatin beads continued to darken, reflecting further moisture absorption, whereas HPMC beads showed a slower progression. By 8 and 24 h, both capsule types exhibited predominantly brown beads, though gelatin capsules showed a darker shade, indicating greater moisture uptake.

### 3.3. Hardness Measurements Results

The results of the capsules’ hardnesses are summarized in Table 2. Gelatin capsules showed a significant reduction in hardness, with a sharp decline after 4 h, suggesting rapid plasticization and softening due to moisture uptake. In contrast, HPMC capsules exhibited a more gradual decrease in hardness, indicating a slower, more controlled response to the humidity.

### 3.4. Moisture Content Results

Thermal analysis was used to assess the moisture uptake in gelatin and HPMC capsules. As shown in Table 2, both capsule types showed an initial increase in water content, with gelatin consistently reaching higher values than HPMC. This is consistent with their respective loss on drying (LOD) values: 14.7% for gelatin and 4% for HPMC according to their manufacturer’s certificate of analysis. Both capsule types exhibited fluctuations in moisture content during the early time points. Moreover, both capsules displayed a dip in moisture content around the 8 h mark, followed by an increase until 24 h. This pattern suggests that moisture absorption is not strictly linear and may involve dynamic interactions between the capsules’ material and the environmental humidity, such as partial moisture loss or redistribution before reaching a new equilibrium. Overall, the gelatin capsules absorbed more moisture than HPMC under these conditions, indicating differing moisture uptake behavior between the two materials.

### 3.5. Surface Wettability Results (Sessile Drop Method)

**Figure 5:**
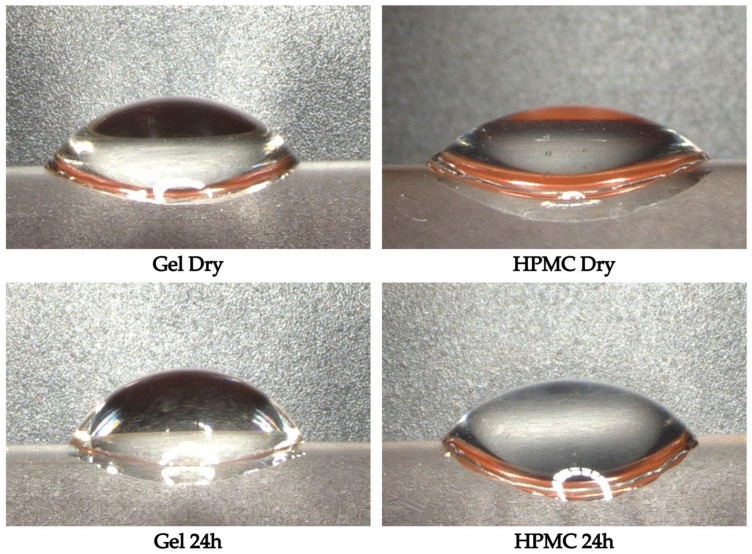
Microscope images showing the sessile drop on gelatin and HPMC capsules in the dry state and after 24 h of exposure to 25 °C, 75% RH conditions.

Sessile drop measurements were performed on gelatin and HPMC capsules to evaluate potential changes in surface wettability following 24 h of exposure to the controlled humidity conditions. The contact angle, representing the degree of surface hydrophilicity, was used as an indicator of how the capsule surface interacts with water before and after moisture uptake. The microscopic images of the sessile drops are shown in Figure 5. Additionally, the contact angle values for both capsule types in the dry and 24 h exposed states, including standard deviations and *p*-values, are provided in Table 3.

In general, contact angle values below 90° are indicative of hydrophilic surfaces. Angles between 30° and 70° are usually considered to show moderate wettability, meaning the surface allows some water to spread but not spread out completely. Lower values, such as those observed for HPMC capsules, suggest a more hydrophilic character, while higher values, like those of gelatin, indicate less wettable but still hydrophilic surfaces.

For gelatin capsules, the average contact angle increased from 53° in the dry state to 60° after 24 h of humidity exposure. This increase was statistically significant (*p* = 0.01), suggesting that the gelatin capsule surface became slightly more hydrophobic after moisture exposure. However, despite this change, the surface remained within the range of moderate wettability. The increase suggests a trend toward reduced hydrophilicity, but not to an extent that would reclassify the surface as hydrophobic. In the case of HPMC capsules, the average contact angle increased from 44° in the dry state to 49° after 24 h. While this shows an upward trend, the difference was not statistically significant (*p* = 0.06). This suggests that the surface wettability of HPMC capsules remained largely unchanged despite moisture uptake.

Notably, the absolute values of the contact angles confirm that both capsule types remained within the hydrophilic range and retain their original wettability characteristics, with HPMC showing consistently lower angles than gelatin, indicating a slightly more wettable surface.

### 3.6. Mechanical Testing Results

#### 3.6.1. Horizontal and Vertical Deformations Tests Results

**Figure 6:**
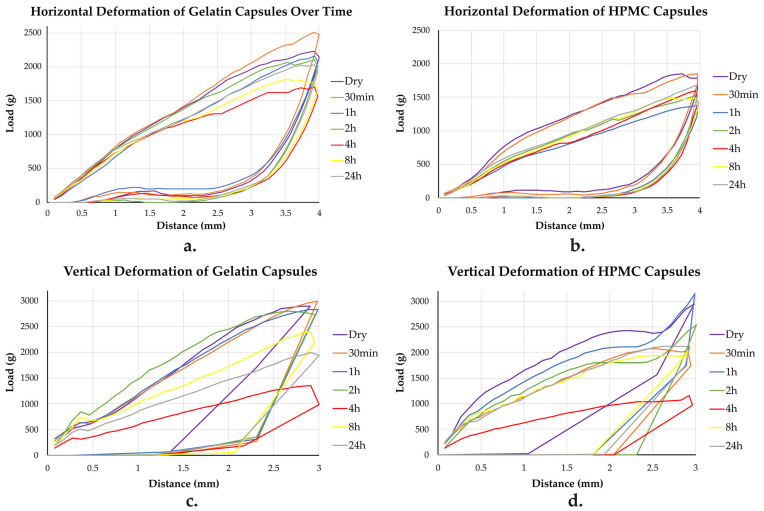
Horizontal (**a**,**b**) and vertical (**c**,**d**) deformations of gelatin and HPMC capsules, when dry and at different time points, after exposure to 25 °C and 75% RH.

To assess how capsule stiffness and structural integrity evolve under humid conditions, horizontal and vertical deformations behavior was examined for both gelatin and HPMC capsules. The corresponding load (g) versus deformation (mm) curves are shown in Figure 6, with the maximum load values summarized in Table 2.

Additionally, Table 4 presents the stiffness and AUC values for both deformations at each time point.

In the horizontal deformation, both capsule types initially exhibited high stiffness and mechanical strength. Upon humidity exposure, they progressively softened, but HPMC exhibited a sharper early decline, particularly by 1 and 4 h. While gelatin showed a steady weakening with gradual partial recovery after 4 h, HPMC displayed more dynamic behavior: initial softening, minor strengthening at 2 and 4 h, but a continued lower mechanical resistance overall. By 24 h, both capsule types showed partial recovery, with gelatin demonstrating a more constant improvement in stiffness, while HPMC’s stiffness and structural properties stabilized with only slight increases. Throughout the 24 h period, HPMC consistently required lower forces to deform compared with gelatin.

The vertical deformation testing showed that gelatin capsules initially had higher stiffness and resistance than HPMC capsules. Upon humidity exposure, HPMC capsules first stiffened at 1 h before softening sharply by 4 h, while gelatin capsules showed a more gradual weakening. At 4 h, both capsule types exhibited their lowest mechanical strength. Gelatin capsules partially recovered by 8 h but remained weaker than they initially were, while HPMC capsules softened further before recovering closer to their original stiffness by 24 h. Overall, HPMC showed a more dynamic but reversible mechanical response, whereas gelatin underwent a more progressive and less reversible weakening.

#### 3.6.2. Puncture Test Results

**Figure 7:**
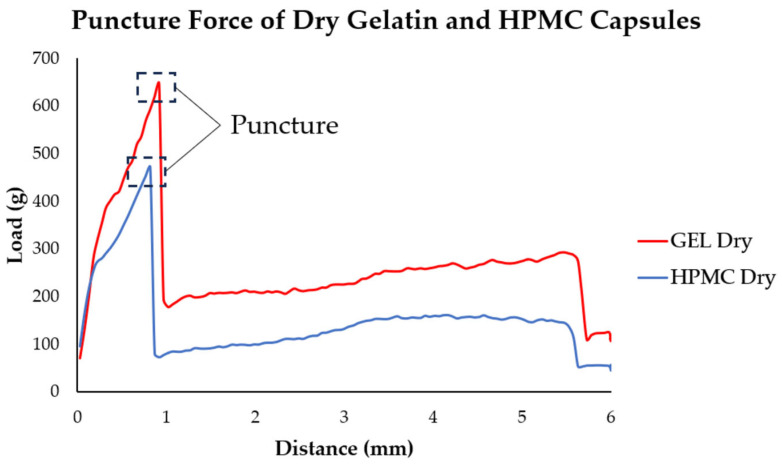
Puncture force of dry gelatin and HPMC capsules. The peak force indicates the point of capsule perforation.

For the puncture strength of gelatin and HPMC capsules, the peak puncture force values are provided in Table 2, with Figure 7 showing the point of capsule perforation where the peak force was recorded.

The puncture testing showed that dry gelatin capsules initially had a higher puncture force than HPMC capsules. Upon humidity exposure, gelatin capsules lost strength rapidly within the first hour but then partially recovered and stabilized up to 8 h before slightly declining again. HPMC capsules showed a delayed decrease, fluctuated more across the earlier time points, and then ultimately had a more pronounced decline by 24 h. Overall, gelatin capsules started stronger but weakened faster, while HPMC capsules exhibited a more variable yet somewhat more stable puncture behavior over time.

### 3.7. PALS Analysis Results

PALS analysis was used to assess the free volume properties of the gelatin and HPMC capsules. The parameters analyzed included the o-Ps lifetime, intensity, and mean lifetime, which provided insights into the polymers’ microstructure changes as a consequence of moisture uptake.

**Figure 8:**
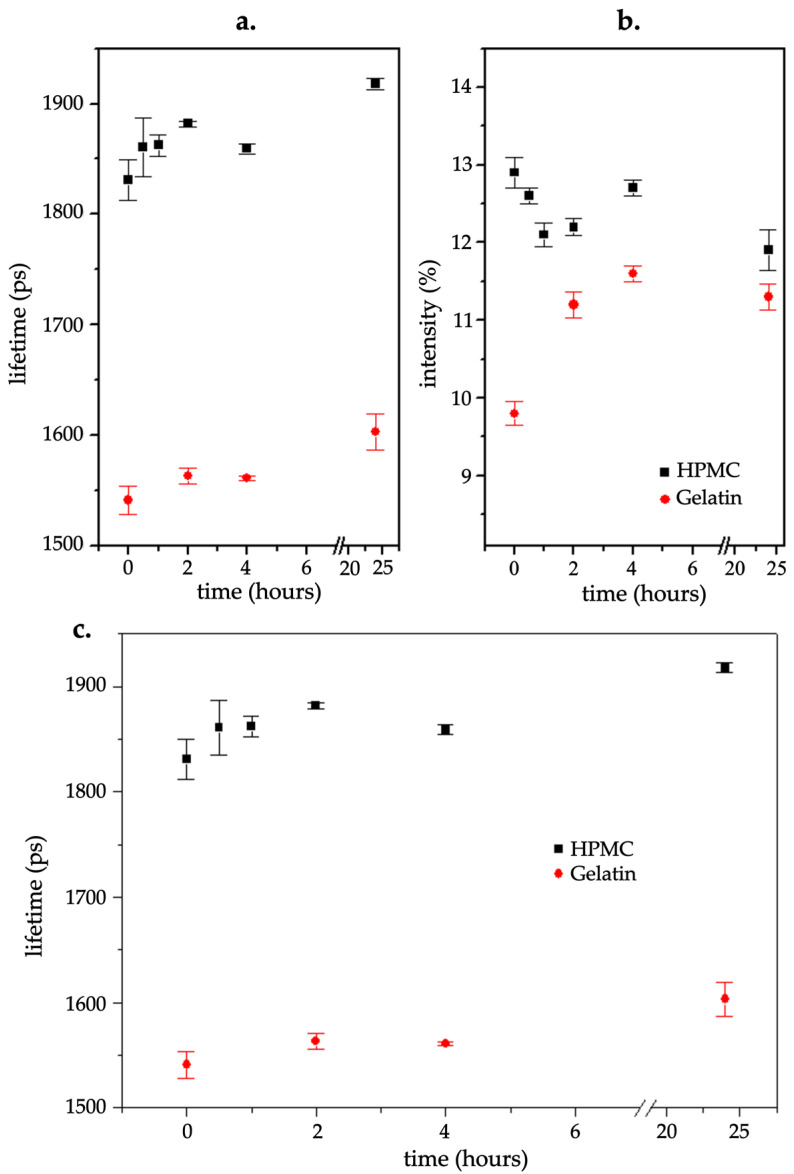
(**a**) o-Ps lifetime, (**b**) o-Ps intensity, and (**c**) mean lifetime of gelatin and HPMC capsules over time during storage at 25 °C and 75% relative humidity. The o-Ps lifetime reflects changes in free volume, the o-Ps intensity indicates variations in the number of o-Ps formation sites, and the mean lifetime represents overall positron annihilation behavior over a 24 h period. Error bars represent the standard deviation of replicate measurements at each time point.

In the case of the o-Ps lifetime, HPMC capsules exhibited considerably higher values compared with gelatin capsules (Figure 8a). The lifetime values of HPMC capsules increased from approximately 1830 ps at the start to around 1925 ps at 24 h, indicating an expansion of free volume. However, depending on the storage period, the values fluctuated, suggesting structural adjustments in response to moisture exposure. These fluctuations imply a dynamic response to moisture that could be linked to moisture absorption-induced plasticization or other microstructural modifications in HPMC capsules. In contrast, gelatin capsules showed a relatively stable o-Ps lifetime around 1545–1600 ps until 24 h, with continuous slight increases observed, pointing to a more gradual but steady structural change.

The intensity of the o-Ps signal, which indicates changes in the number of sites where o-Ps forms, followed a different pattern (Figure 8b). Initially, HPMC capsules had higher intensity values (~12.9%) compared with gelatin (~9.8%). Over time, the HPMC intensity decreased, then increased, before decreasing again at 24 h, while gelatin intensity increased until 4 h and then decreased by 24 h. At the 24 h mark, the intensities converged at approximately 11.3% for gelatin and 11.8% for HPMC.

The mean lifetime values (Figure 8c) for HPMC capsules fluctuated over the initial hours of exposure before steadily increasing between 1 and 4 h, around 460–475 ps. However, at 24 h, a decrease was observed to a value of approximately 455 ps. Alternatively, gelatin capsules started at a lower mean lifetime (~377 ps), then increased gradually, reaching ~420 ps at 24 h, reflecting a progressive expansion of free volume as moisture uptake progressed.

Overall, the PALS results highlighted the differences in the moisture response between HPMC and gelatin capsules. HPMC capsules exhibited greater fluctuations in free volume parameters, suggesting a more dynamic molecular rearrangement in response to moisture, while gelatin capsules demonstrated a more gradual and less pronounced modification.

## 4. Discussion

### 4.1. Initial Phase—Early Stability Response (Dry and 30 min Exposure)

The initial phase captures the onset of capsule–moisture interactions. At 0 h, both gelatin and HPMC capsules were in a dry state, but after 30 min, early moisture uptake was evident through mass increase. HPMC capsules, with lower initial moisture content, showed a greater percentage mass increase than gelatin, suggesting that they absorb moisture more efficiently under high humidity. This could be due to HPMC’s higher hydrophilicity [25] and polymer structure allowing greater water uptake through interactions between the polymer chains and moisture in the environment [26]. As the color change of silica beads in gelatin capsules is more pronounced, the results suggest differing patterns of moisture absorption and distribution: HPMC likely absorbs moisture more efficiently at the surface, while gelatin allows for faster internal penetration.

In terms of mechanical properties, gelatin capsules initially exhibited higher hardness and puncture resistance, indicating greater rigidity compared with HPMC. However, after 30 min, both capsule types softened.

At the start, both gelatin and HPMC capsules showed minimal horizontal deformation, which is typical for dry capsules. However, after 30 min of moisture exposure, the horizontal deformation and puncture increased more in gelatin, aligning with softening, whereas HPMC showed a minimal deformation change, indicating a more gradual mechanical response despite higher moisture uptake.

The vertical deformation showed gelatin’s higher initial stiffness and HPMC’s more elastic structure. However, after 30 min, HPMC showed early softening, suggesting that HPMC is more sensitive to moisture uptake, which likely begins to disrupt its polymer structure. This points to a rapid interaction between water and the HPMC matrix, even in short exposure durations.

Thermal analysis confirmed gelatin’s higher initial moisture and greater increase after 30 min, while HPMC absorbed moisture more rapidly at first. The higher moisture in gelatin likely lowered its glass transition temperature (Tg), facilitating softening, unlike the more gradual structural response in HPMC.

Additionally, the PALS results provided valuable insights into the microstructural changes occurring within the capsules as they absorb moisture. They revealed lower initial o-Ps lifetime values in gelatin, consistent with the higher initial moisture content, rigidity, and resistance to puncture in the dry state. The higher initial o-Ps lifetime in HPMC capsules suggested greater initial free volume, likely linked to their hydrophilic nature and capacity for rapid moisture uptake. After 30 min, HPMC’s increased o-Ps lifetime indicated rapid moisture-induced structural expansion. However, this did not translate into significant horizontal deformation, indicating that the moisture uptake did not lead to significant softening of the capsule wall at this stage. In contrast, gelatin showed a gradual increase coupled with greater horizontal deformation—suggesting earlier softening despite lower moisture uptake. In terms of PALS intensity, HPMC capsules showed a higher intensity at 0 h, indicating a higher number of available free volume sites for positron annihilation. After 30 min, the intensity decreased slightly, suggesting that moisture absorption led to rearrangements in the polymer structure, reducing the number of available sites for o-Ps formation. On the contrary, the intensity in gelatin capsules seemed to be increasing gradually during the earlier time points, reflecting an increase in the number of free volume sites as the capsules absorbed moisture.

In summary, within 30 min, gelatin capsules softened more visibly despite lower water uptake, while HPMC absorbed more moisture but showed subtler mechanical changes, reflecting distinct moisture absorption and structural responses.

### 4.2. Intermediate Phase—Progressive Stability Response (1 h to 4 h)

Between 1 and 4 h, moisture uptake continued but followed distinct patterns.

From 1 to 2 h, HPMC’s moisture uptake slowed down, approaching saturation of the material’s moisture-binding sites, then stabilized. Gelatin, on the other hand, continued its steady, gradual absorption.

The silica beads’ color changes supported these observations. HPMC capsules showed early signs of browning that progressed slowly, indicating a possible surface saturation. Gelatin capsules transitioned more consistently and rapidly to brown.

The hardness decreased in both capsules, with HPMC softening rapidly between 1 and 2 h before stabilizing, while gelatin showed a more gradual hardness decrease followed by a sharp drop from 2 to 4 h, correlating with the beads’ color changes.

The moisture content analysis provided further insights into the structural changes occurring within the capsules. In HPMC capsules, the moisture content initially decreased, suggesting near-saturation. However, a sharp increase was then observed, consistent with the silica beads’ color change, which could indicate that the HPMC capsules began absorbing moisture again at a more pronounced rate as moisture may have reached deeper layers of the capsule or as surface moisture was retained. In contrast, gelatin capsules showed a continuous decrease despite a steady mass increase during this period. These changes suggest that structural changes within the gelatin matrix may have allowed some moisture to evaporate or escape. It is possible that the gelatin matrix softened or swelled as it absorbed moisture, leading to the loss of some moisture from the capsule itself. Meanwhile, the silica beads inside the capsule continued to absorb moisture, as evidenced by their color change. This discrepancy could be due to the beads not being affected by the changes in the gelatin structure, allowing them to retain moisture while the capsule lost some.

Regarding mechanical testing, gelatin softened in both horizontal and vertical deformation tests, with the weakest load observed at 4 h (similarly to HPMC). HPMC capsules, on the other hand, exhibited contrasting trends between horizontal (softening then partial recovery) and vertical (initial stiffness then gradual softening) deformations. Based on these findings, it seems that the moisture penetration into the HPMC and gelatin capsules occurred differently. In HPMC capsules, the moisture initially penetrated the walls of the capsule, causing them to soften first. As time passed, moisture gradually spread deeper into the structure, eventually reaching the domes of the capsule. This process resulted in the differences in mechanical responses (horizontal vs. vertical deformation), with the walls softening earlier than the domes. In contrast, it seems that the gelatin capsules absorbed moisture more evenly throughout the entire capsule. As moisture steadily entered the gelatin matrix, it caused a gradual weakening of the capsule’s structure. The moisture uptake seemed to affect both the walls and domes similarly, leading to a more consistent decrease in load during both horizontal and vertical mechanical testing.

The puncture tests revealed that gelatin briefly strengthened then stabilized, possibly indicating that, despite the continued moisture absorption, the gelatin matrix began to adjust structurally, possibly due to the capsule absorbing moisture more evenly. For HPMC, the puncture strength fluctuated, indicating possible surface evaporation or redistribution. These findings suggest a shift towards equilibrium, where moisture absorption continued more gradually while surface moisture dynamics changed. The changes in puncture strength at 1 h, alongside the vertical deformation data, also imply that the capsule walls were affected earlier by moisture than the domes, which likely absorbed moisture later.

The PALS analysis revealed that for gelatin capsules, the o-Ps lifetime remained mostly stable, suggesting minimal fluctuation in free volume. This aligns with the gradual softening and increased mass observed in gelatin capsules during the same period. In contrast, the o-Ps lifetime for HPMC capsules increased gradually from the start to 2 h, indicating that moisture absorption led to an increase in free volume. However, after 2 h, the o-Ps lifetime decreased, reflecting deeper moisture penetration and slower absorption, suggesting a shift in internal structure as the capsule became potentially more stable. The o-Ps intensity for gelatin capsules increased steadily from the start, aligning with the gradual softening and increased mass observed in gelatin capsules during the same period. For HPMC capsules, on the other hand, intensity decreased early on, likely due to surface moisture loss or redistribution, before increasing from 1 to 4 h, suggesting more consistent moisture absorption and greater interaction with the capsule material as moisture penetrated deeper into the walls. The mean o-Ps lifetime for gelatin capsules gradually increased, consistent with mechanical softening, while for HPMC capsules, it decreased initially, likely due to early moisture absorption, then increased as the structure stabilized.

In summary, between 1 and 4 h, gelatin capsules showed steady moisture uptake with progressive softening, while HPMC capsules exhibited rapid initial absorption followed by structural stabilization. PALS analysis confirmed consistent softening in gelatin and more dynamic molecular changes in HPMC, reflecting their distinct moisture response behaviors.

### 4.3. Final Phase—Late Stability Response (8 h to 24 h)

In the final phase, prolonged exposure to humidity accentuated the differences in capsule behavior, offering insights into their long-term stability. Both capsule types absorbed substantial moisture, but their structural responses diverged significantly.

Gelatin capsules’ mass gain plateaued after 8 h, suggesting a possible saturation. In contrast, HPMC capsules exhibited a temporary mass decrease between 4 and 8 h, which could be attributed to surface-level moisture redistribution. It has been shown that HPMC capsules exhibit dynamic moisture behavior: findings from low-field magnetic resonance imaging (LF-MRI) studies show that moisture within HPMC shells can migrate from the interior to the surface, promoting dynamic interactions with the surrounding environment. HPMC capsules have been found to have lower moisture sorption and retention capabilities compared with gelatin capsules, which can result in more a pronounced moisture loss or redistribution under certain conditions [27].

Silica bead color changes aligned with the previous findings. In gelatin capsules, beads were fully darkened by 8 h and remained unchanged, indicating steady moisture uptake. In HPMC capsules, the darkening of the beads was slower and more variable. This contrast can be linked to the differing moisture uptake behaviors of the two capsule types. Gelatin is known to have a stronger affinity for moisture and a different mechanism of water absorption compared with HPMC, as demonstrated by Braham et al., who reported a greater moisture attraction in gelatin capsules and observed crystallization of spray-dried lactose when RH increased from 50% to 70% [28].

The gelatin capsules softened substantially by 8 h, which likely reflects the moisture uptake and subsequent physical changes occurring within the gelatin matrix. Moisture-induced plasticization is also associated with a decrease in the material’s Tg, which marks the transition from a rigid to a rubbery state. A lowered Tg facilitates molecular mobility within the polymer chains and thus enhances softness and deformability of the material. This behavior is commonly observed in hydrophilic polymers such as Eudragit, where the addition of plasticizers or moisture decreases intermolecular interactions between polymer chains, thus improving flexibility and deformability [29]. Similarly, Chiwele et al. observed that gelatin capsules stored under tropical humid conditions (37 °C, 75% RH) for 24 h exhibited significant softening and stickiness, indicating a clear impact of moisture on the capsule’s structural integrity [30]. Likewise, another study by Pinto et al. showed that gelatin capsules exhibited increased tribo-charging at low RH, highlighting gelatin’s sensitivity to moisture changes [31]. In contrast, the HPMC softened less. Chiwele et al. similarly noted that HPMC capsules tend to maintain greater structural stability in the presence of moisture, likely due to their lower hygroscopicity and the stronger interactions between the HPMC polymer chains [30].

The moisture content results further support the observations regarding moisture dynamics in both capsule types: both showed decreased moisture content between 4 and 8 h. For HPMC, this decline coincided with the observed mass drop, consistent with surface redistribution and minor moisture loss. For gelatin capsules, however, the mass continued to increase during this same time frame, indicating that despite the moisture content reduction, the capsules were still absorbing moisture from the environment. This aligns with findings that gelatin capsules exhibit higher moisture uptake across various relative humidity conditions compared with HPMC capsules, as demonstrated by Barham et al. [28]. Additionally, Wang et al. reported that HPMC capsules have weaker moisture sorption and retention capabilities than gelatin capsules, which further supports the observation that HPMC’s moisture absorption behavior is more dynamic. From 8 to 24 h, both capsule types showed a similar pattern of increased moisture content, reflecting a stabilization in moisture uptake. The levels of moisture retained by each capsule type were still distinct, emphasizing the differing absorption behaviors observed earlier. This pattern suggests that while both capsule types ultimately equilibrate in terms of moisture absorption rate, their individual capacity for moisture retention and the mechanisms behind this uptake remain divergent.

The sessile drop measurements revealed a significant contact angle increase in gelatin, suggesting a reduction in surface wettability and an increase in hydrophobicity. The increase in contact angle is likely due to the formation of a more hydrophobic surface layer or a change in the capsule’s surface morphology due to moisture-induced plasticization. These changes in surface properties may reduce the surface energy of the capsule, as moisture exposure can lead to rearrangements of the polymer chains, causing hydrophilic groups to become less exposed on the surface, making the capsule more hydrophobic. This phenomenon has been observed in various polymer systems, where hydration induced reorganization of surface segments, leading to changes in wettability [32,33]. Contrastingly, HPMC capsules showed only a minor, non-significant contact angle increase, reflecting their more stable hydrophilic surface and lower sensitivity to moisture-induced surface changes.

The horizontal deformation tests showed distinct mechanical responses. Gelatin capsules initially softened then partially stiffened laterally, suggesting moisture-induced polymer network reorganization. As the material approached a possible saturation, polymer chains likely re-established intermolecular interactions such as hydrogen bonding, leading to stronger or denser structural arrangements, which limited further deformation. These observations are consistent with similar findings reported for gelatin capsules exposed to 11, 22, and 51% RH, where an initial softening due to moisture uptake was followed by stabilization, likely related to structural reorganization [31]. However, the 24 h values remained below earlier points, indicating that the gelatin structure never fully regained its initial mechanical strength. In comparison, HPMC capsules exhibited a more gradual and stable mechanical response, with mild softening followed by stabilization. This aligns with the behavior of HPMC capsules exposed to various RH levels (11, 22, and 51%), which exhibited only moderate changes in their electrostatic response, further supporting HPMC’s overall structural stability in the presence of moisture [31].

The vertical deformation tests revealed further divergence. The gelatin capsules stiffened then softened, suggesting that while the domes initially became more resistant to compression, possibly due to localized thickening or stiffening, they later became more compliant again, likely as a result of a cumulative plasticization. This pattern reflects a non-uniform response within the capsule: while the lateral walls consolidated, the dome region may have become more susceptible to moisture-induced softening over time. HPMC capsules, however, showed a progressive strengthening of the dome region. The relatively mild changes observed are consistent with surface wettability findings, highlighting the greater structural resilience of HPMC under moisture stress.

For gelatin, in terms of puncture force, the values remained relatively stable. This mirrors the previously noted structural behavior of the gelatin capsules: even though they underwent significant softening and mass increase earlier in the exposure period, the puncture resistance appears to have stabilized once the matrix began reorganizing. HPMC capsules, in contrast, showed intermediate increases and decreases in resistance, which correspond well with the increase in load seen in the vertical deformation, while the following decline could reflect the capsule’s adjustment to prolonged moisture exposure, with minor relaxation of the polymer network as an equilibrium was approached. This correlates with the relatively steady contact angle values and the stability observed in the mechanical stiffness, reinforcing the interpretation of HPMC’s dynamic but overall stable structural response under humid conditions. Interestingly, this stability was also observed even at lower humidity levels (11% and 33% RH), where the puncture properties of HPMC capsules were less affected than gelatin capsules [34].

Regarding the PALS results, for the gelatin capsules, the o-Ps lifetime gradually increased throughout the exposure period, with a significant rise at 24 h. This suggests an increase in free volume and molecular rearrangement, typically associated with moisture absorption, which likely promotes polymer chain movement and results in a more flexible structure. As the gelatin matrix absorbed moisture, plasticization of the polymer chains likely occurred, enabling the formation of additional free-volume pockets within the matrix. This could explain the observed rise in o-Ps lifetime, as ortho-positronium atoms become more trapped within these expanded regions. The reduction in o-Ps intensity at 24 h further reflects a shift in the material’s microstructure, where the increased free volume led to fewer positron annihilation occurrences, a typical result of plasticization and matrix softening. In contrast, HPMC capsules showed an increase in o-Ps lifetime over the same period, which could be indicative of gradual moisture-induced changes in the HPMC polymer network. This behavior is consistent with HPMC’s lower hygroscopicity and more stable structural response to moisture, as previously observed. Interestingly, the o-Ps intensity for HPMC capsules decreased at 24 h, aligning closely with the gelatin capsules’ intensity at this time point. This decrease suggests that, while the HPMC matrix did not undergo the same level of plasticization as gelatin, the prolonged moisture exposure may have still led to changes in its microstructure, allowing for the formation of free-volume pockets, although to a lesser extent. The decrease in intensity for both gelatin and HPMC could reflect a dynamic equilibrium between moisture absorption and the molecular rearrangements occurring within the polymer matrix. While both capsule types exhibited an increase in free volume over time, gelatin capsules demonstrated more pronounced molecular mobility, corresponding with their higher moisture retention and softer, more plasticized structure. In contrast, HPMC capsules showed a more gradual, stable response, with slower molecular rearrangements, consistent with their lower moisture sorption and more stable mechanical behavior.

### 4.4. Experimental Approach and Relevance to DPI Performance

The experimental design in this study aimed to provide a comprehensive understanding of how gelatin and HPMC capsules respond to moisture exposure. By combining physical, mechanical, and molecular-level analyses, we aimed to evaluate their robustness under conditions that simulate realistic storage environments, thereby assessing their suitability for DPI applications. A thorough understanding of the relationship between capsule properties, formulation characteristics, device design, and patient-specific factors is essential for developing robust manufacturing processes and achieving consistent drug delivery to the lungs [35].

The mass measurements served as a primary indicator of the extent of the moisture uptake, which allowed for a quantification of the absorbed moisture and its potential influence on capsule integrity and ability to protect formulations from moisture. As moisture uptake can compromise both the capsule structure and drug stability, mass changes offer a straightforward metric for DPI suitability [10].

The visual observation of silica beads color changes offered an illustrative and qualitative indication of the internal humidity protection and how effectively each capsule type shielded its contents from ambient humidity—an essential factor for preserving powder stability and ensuring dose consistency in DPI formulations.

Moreover, changes in structural integrity were investigated by hardness testing, which revealed early signs of softening as capsules absorbed moisture. This is particularly critical in DPI applications, where capsules must be strong enough to withstand piercing without cracking or collapsing, as such defects can hinder dose delivery.

In addition, moisture content analysis confirmed internal water absorption—which can alter flexibility and stickiness—both of which can hinder proper device needle piercing and efficient powder release. These results reinforced the mass data and offered a more detailed picture of the internal moisture uptake. This type of analysis is particularly valuable given that differences in sorption behavior between capsule materials, such as gelatin and HPMC, can significantly influence their ability to preserve formulation stability under humid conditions [28].

The contact angle measurements assessed surface wettability changes, which is a particularly important characteristic for DPIs, where capsule–wall interactions with the powder formulation can influence how efficiently the dose is released during inhalation. An increase in contact angle indicates reduced surface hydrophilicity, which may lead to weaker adhesion between the capsule and hygroscopic powders. Inversely, if the surface becomes more hydrophilic, powders may stick to the capsule wall, reducing the emitted dose [36,37]. From a practical standpoint, even when a change in contact angle is not statistically significant—as we observed for HPMC capsules—absorbed moisture can still plasticize and weaken the capsule wall. Though HPMC capsules showed a less significant increase in contact angles, the absorbed moisture could still impact their internal properties and affect the capsule’s ability to be properly punctured. Because DPI capsules must be perforated adequately by the inhaler device to ensure efficient aerosolization, any moisture-induced softening could compromise puncture quality and ultimately reduce the delivered dose.

Mechanical testing was used to evaluate whether moisture exposure caused softening or weakening of the capsule shell. Each test targeted specific mechanical aspects: horizontal deformation assessed wall flexibility; vertical deformation focused on the dome, typically pierced in DPIs; and puncture testing simulated the actual needle insertion process [19,38]. Together, these tests captured both localized and overall mechanical behavior, helping to characterize the extent to which capsule softening may impact DPI functionality.

To interpret the PALS results, it is important to first consider the concept of polymer free volume. Polymer free volume refers to the unoccupied space within a polymer matrix not filled by polymer chains or other molecular components [39]. First formalized by Cohen and Turnbull in 1961, it represents the portion of thermal expansion that can be redistributed without an energy change [40]. Free volume is a consequence of imperfect polymer chain packing, predominantly in amorphous regions characterized by disordered molecular arrangements that generate voids [41]. This concept is closely tied to the intrinsic structure of pharmaceutical polymers. Gelatin exhibits unique free volume characteristics due to its protein-based structure and semi-crystalline nature. The polymer consists of polypeptide chains with complex secondary structures including helical regions and random coils [42]. This structural complexity results in a heterogeneous free volume distribution, with different environments for water binding and molecular transport. The free volume in gelatin is strongly influenced by moisture content, as water molecules can interact with hydrophilic amino acid residues and become incorporated into the polymer matrix [42]. This leads to significant changes in free volume distribution and mechanical properties, as demonstrated by the PALS measurements showing gradual increases in o-Ps lifetime with moisture exposure.

In contrast, HPMC shows a different polymer architecture: it is a cellulose derivative polymer characterized by a backbone of glucose units substituted with hydroxypropyl and methyl groups, which significantly affect its hydrophilicity and free volume properties [43]. The amorphous nature of HPMC results in a more uniform free volume distribution compared with gelatin. HPMC exhibits dynamic free volume behavior under moisture exposure, with the PALS measurements revealing fluctuating o-Ps lifetimes that suggest molecular rearrangement and adaptation. This behavior reflects the polymer’s ability to accommodate moisture through structural reorganization rather than a simple cavity filling. Ultimately, polymer free volume is a key property influencing DPI capsule performance, and its measurement via techniques such as PALS reveals how intrinsic structural differences, such as those between gelatin and HPMC, affect moisture response.

Our PALS measurements provided molecular-scale insights by assessing changes in the free volume within capsule materials. A decrease in free volume implies denser molecular packing, which may translate to an increased brittleness or reduced flexibility of the capsule. PALS provided a deeper understanding of how moisture affects the internal structure of the capsules beyond what is observable at the macroscopic level by mechanical or physical tests. By monitoring changes in the energy of annihilation photons, PALS can probe the local electron–momentum distribution in the material, providing complementary insights into its molecular structure and microstructural changes [44]. In particular, PALS allowed for the detection of changes in free volume associated with moisture absorption, revealing molecular-level alterations that might not be captured through conventional mechanical testing methods. These insights are particularly relevant in polymeric systems, where water can influence the physical properties of the material and its overall stability [45], and ultimately affect how easily a DPI capsule can be pierced and its powder released.

While the fundamental differences between these capsule materials are well established, this study introduces several novel contributions that advance capsule characterization under humidity stress beyond the existing literature. Most notably, the most significant methodological innovation is the application of PALS to monitor free volume changes in gelatin and HPMC capsule shells during moisture exposure. While PALS has been used in pharmaceutical applications for examining microstructures and monitoring changes in various drug delivery systems [45], its specific application to capsule humidity response represents a new frontier.

Moreover, the use of silica gel beads as internal moisture indicators and the application of sessile drop contact angle analysis to capsule surfaces before and after exposure also represent novel assessment methods. While silica gel beads are commonly used as desiccants, their application as real-time visual indicators of moisture penetration within capsule shells has not been previously reported in the pharmaceutical literature. In addition, while contact angle measurements are well established for assessing the wettability of pharmaceutical powders, their application to capsule materials (especially before and after exposure to humidity) has not been documented. The observation that gelatin capsules show significant contact angles increase after humidity exposure provides new insights into surface reorganization mechanisms.

Overall, the systematic integration of a broad set of analytical tools enabled correlations between molecular and macroscopic behaviors, contributing to a deeper understanding of capsule performance, with important implications for formulation development and product optimization.

Altogether, the combined findings (summarized in Table 5) from physical measurements, mechanical testing, and molecular-scale analysis offer a multidimensional perspective of how gelatin and HPMC capsules behave under humid conditions. These findings not only support informed capsule selection for DPI formulations but also emphasize the broader importance of addressing environmental sensitivity in the design and development of drug delivery systems.

All in all, the HPMC capsules demonstrated greater resilience to humidity; although they absorb moisture more rapidly, they exhibit dynamic adaptation at the molecular level, retaining better mechanical integrity and surface properties. In contrast, gelatin capsules—despite their initially higher strength—undergo pronounced softening and a marked decline in puncture resistance after moisture exposure, with less capacity for a possible structural recovery. These differences support the suitability of HPMC capsules for DPI formulations intended for use in humid environments.

## 5. Conclusions

In conclusion, this study highlighted the significant effects of environmental humidity on the structural and mechanical behavior of gelatin and HPMC capsules used in DPIs. Gelatin capsules absorbed more moisture and underwent notable plasticization over time, leading to changes in mechanical properties. In contrast, HPMC capsules exhibited a more gradual and stable response, with limited structural changes and only moderate variation in mechanical behavior. PALS analysis confirmed a more pronounced increase in free volume for gelatin, suggesting greater molecular mobility under humid conditions, whereas HPMC showed a slower, more subtle rearrangement of its polymer network. These findings suggest that HPMC capsules may provide superior mechanical and structural stability, as well as performance under humid conditions compared with gelatin capsules, making them a preferable choice for DPI applications in environments prone to high humidity. To support such applications, existing quality standards could benefit from being expanded to include puncture resistance testing for and stability studies that expose DPI capsules to high-humidity conditions, accompanied by mechanical evaluations to ensure functionality under real-world moisture stress. Future studies should explore the clinical implications of these material properties, focusing on their influence on lung deposition patterns and their potential impact on patient adherence.

## Figures and Tables

**Table 1:** Summary of the mechanical tests parameters using a TexturePro Texture Analyzer.

Test Type	Capsule Orientation	Probe Type	Probe Diameter (mm)	Probe Length (mm)	Test Speed (mm/s)	Target Distance (mm)	Purpose
Horizontal deformation	Horizontal	TA5 cylinder	12.7	35	1.00	4.0	Measures deformation from side compression; assesses lateral structural integrity.
Vertical deformation	Vertical	TA5 cylinder	12.7	35	1.00	3.0	Evaluates capsule’s vertical deformation response under downward pressure.
Puncture	Horizontal	TA9 needle	1.0	35	0.50	6.0	Measures force required to puncture the capsule.

**Table 2:** Comparison of the main physical, mechanical, and structural parameters for gelatin and HPMC capsules at each time point under 25 °C, 75% relative humidity (RH).

Time Point	Capsule Type	Mass Gain (%)	Moisture Content (%)	Hardness (N)	Max. Horizontal Deformation Force (g)	Max. Vertical Deformation Force (g)	Max. Puncture Force (g)	o-Ps Lifetime ^1^ (ps)	o-Ps Intensity (%)	Mean Lifetime ^1^ (ps)
0 h (dry)	Gelatin	0	10.33 ± 0.41	60 ± 5.6	2230.0	2897.4	582.1 ± 59.1	~1545	~9.8	~377
	HPMC	0	6.27 ± 0.27	57 ± 5.4	1845.0	2947.3	525.5 ± 89.1	~1830	~12.9	~457
30 min	Gelatin	0.78 ± 0.4	11.60 ± 0.37	-	2506.6	2993.1	519.1 ± 79.1	-	-	-
	HPMC	2.25 ± 0.5	6.37 ± 0.11	-	1846.8	2073.3	465.5 ± 69.7	↑	↓	↑
1 h	Gelatin	1.12 ± 0.5	13.36 ± 0.45	64 ± 3.4	2157.1	2831.7	383.9 ± 54.0	-	-	-
	HPMC	4.15 ± 0.5	8.03 ± 0.74	44 ± 0.4	1385.6	3149.1	496.8 ± 51.9	stable	↓	↓
2 h	Gelatin	1.61 ± 0.5	13.00 ± 0.21	56 ± 4.1	2103.1	2797.3	534.7 ± 60.0	↑	↑	↑
	HPMC	4.41 ± 0.6	7.83 ± 0.08	45 ± 0.4	1531.2	2536.8	388.4 ± 37.6	↑	↑	↑
4 h	Gelatin	2.67 ± 0.6	14.82 ± 0.96	22 ± 1.5	1701.5	1351.6	555.4 ± 76.0	stable	↑	↑
	HPMC	5.03 ± 0.4	6.60 ± 0.28	41 ± 2.9	1592.7	1157.4	423.4 ± 42.1	↓	↑	↑
8 h	Gelatin	3.66 ± 0.6	11.07 ± 0.69	25 ± 1.8	1817.1	2406.8	555.1 ± 27.5	-	-	-
	HPMC	4.30 ± 0.8	5.53 ± 0.20	43 ± 4.4	1490.4	2076.3	493.1 ± 26.8	-	-	-
24 h	Gelatin	3.77 ± 0.5	15.05 ± 0.16	-	2029.3	1991.6	506.8 ± 35.6	~1600	~11.3	~420
	HPMC	5.46 ± 0.4	8.78 ± 0.40	-	1676.6	2122.9	411.2 ± 52.0	~1925	~11.8	~455

“↑” and “↓” indicate relative trends compared with previous time points. Empty cells (“-”) indicate data not explicitly provided for that time point. Values are presented as the mean ± SD where applicable. ^1^ The o-Ps lifetime corresponds to the lifetime of ortho-positronium trapped in free volume holes, while the mean lifetime represents the overall average lifetime of positrons annihilating in all environments within the material (free volume regions and denser areas).

**Table 3:** Contact angle values (°) for dry and 24 h exposed gelatin and HPMC capsules, presented as the mean ± standard deviation (SD), with *p*-values included (*n* = 5).

Capsule Type	State	Average Contact Angle (° ± SD)	*p*-Value
Gelatin	Dry	53 ± 4.2	0.01
	24 h exposure	60 ± 2.7	
HPMC	Dry	44 ± 3.2	0.06
	24 h Exposure	49 ± 1.2	

**Table 4:** Stiffness (N/mm) and area under the curve (AUC, N/mm) for gelatin and HPMC capsules dry and stored at various time points, under 25 °C and 75% RH, following horizontal and vertical deformations. Values are presented as the mean (*n* = 5) ± standard deviation (SD) for each time point.

Test	Metric (N/mm)	Capsule Type	Dry	30 min	1 h	2 h	4 h	8 h	24 h
Horizontal deformation	Stiffness	Gelatin	5.71 ± 0.459	6.24 ± 1.320	5.55 ± 0.144	5.34 ± 0.499	4.09 ± 0.305	4.55 ± 0.511	4.96 ± 0.635
		HPMC	4.63 ± 0.871	4.54 ± 1.141	3.40 ± 0.226	3.69 ± 0.912	3.87 ± 0.891	3.92 ± 0.844	3.96 ± 0.746
	AUC	Gelatin	37.6 ± 2.00	42.8 ± 4.62	29.8 ± 2.03	38.7 ± 1.44	31.4 ± 1.20	33.5 ± 1.80	35.4 ± 4.11
		HPMC	34.3 ± 3.17	33.8 ± 2.68	25.4 ± 2.88	28.5 ± 2.95	27.4 ± 3.84	30.0 ± 3.14	30.1 ± 1.90
Vertical deformation	Stiffness	Gelatin	10.36 ± 0.614	9.57 ± 0.244	9.40 ± 1.033	9.52 ± 0.188	7.55 ± 0.097	7.22 ± 0.130	7.44 ± 0.275
		HPMC	7.44 ± 0.244	6.86 ± 0.377	7.76 ± 0.437	6.27 ± 0.320	5.77 ± 0.318	5.72 ± 0.280	7.00 ± 0.826
	AUC	Gelatin	25.8 ± 4.35	25.8 ± 2.61	26.0 ± 2.59	31.2 ± 1.08	22.7 ± 2.83	21.4 ± 2.54	22.5 ± 2.00
		HPMC	23.6 ± 4.09	24.9 ± 1.03	35.1 ± 1.61	30.1 ± 1.87	22.5 ± 1.37	19.7 ± 0.99	22.8 ± 4.90

**Table 5:** Comparative performance overview of gelatin and HPMC capsules.

Property	Gelatin Capsules	HPMC Capsules
Initial water content	Higher (10–14%)	Lower (4–6%)
Moisture absorption	Slower, steady	Rapid, fluctuating
Equilibrium moisture (after 24 h)	Higher	Lower
Hardness changes	Rapid, significant	Gradual, moderate
Puncture resistance	Higher initially, drops	Lower initially, more stable
Hydrophilicity	Moderate, decreases	High, stable
Free volume expansion	Gradual	Greater, dynamic
Mechanical recovery	Partial, slow	Partial, faster

## Data Availability

The data presented in this study are openly available in the article.

## Abbreviations

The following abbreviations are used in this manuscript:

HPMC	Hydroxypropyl methylcellulose
DPI	Dry powder inhaler
COPD	Chronic obstructive pulmonary disease
FPD	Fine particle dose
ICH	International Council for Harmonisation (of Technical Requirements for Pharmaceuticals for Human Use)
PALS	Positron annihilation lifetime spectroscopy
RH	Relative humidity
AUC	Area under the curve
o-Ps	ortho-positronium
LOD	Loss on drying
Tg	glass transition temperature
